# Explicit and implicit self-esteem in youth with autism spectrum disorders

**DOI:** 10.1177/1362361320961006

**Published:** 2020-10-15

**Authors:** Renske van der Cruijsen, Bianca E Boyer

**Affiliations:** 1 Erasmus University Rotterdam, The Netherlands; 2 University of Amsterdam, The Netherlands; 3 Psychologenpraktijk Kuin, The Netherlands

**Keywords:** adolescents, autism spectrum disorders, children, explicit self-esteem, implicit association task, implicit self-esteem

## Abstract

Although the link between self-esteem and psychopathology has been well established, studies on self-esteem in individuals with autism spectrum disorder are lacking. In this study, we aimed to (1) compare explicit and implicit self-esteem of youth with autism spectrum disorder to typically developing peers and to (2) explore relationships of implicit-, explicit-, and discrepant self-esteem measures with co-occurring internalizing and externalizing problems in youth with autism spectrum disorder. For this purpose, 25 individuals with autism spectrum disorder and 24 individuals as age- and intelligence quotient–matched controls aged 8–16 years participated in this study. Results showed lower explicit self-esteem in autism spectrum disorder compared to typically developing youth and no differences in implicit self-esteem between groups. In youth with autism spectrum disorder, low explicit self-esteem was related to co-occurring depression symptoms, whereas lower implicit self-esteem was related to externalizing symptoms. These results show that youth with autism spectrum disorder are at risk for developing low explicit self-esteem, which appears to be related to often co-occurring internalizing symptoms. This emphasizes the need to focus more on self-esteem in assessment and treatment of youth with autism spectrum disorder.

**Lay abstract:**

Having a stable and good self-esteem is important for maintaining a good mental health. However, having low self-esteem is a risk factor for developing depressive, anxious, or uncooperative/aggressive symptoms. While many individuals with an autism spectrum disorder have these symptoms, there is a lack of studies on self-esteem in this group. We studied self-esteem of youth with autism spectrum disorder and the connection to their co-occurring symptoms. To do this, different self-esteem profiles were investigated, including explicit self-esteem (how someone says their self-esteem is after reflecting on it), implicit self-esteem (how someone’s self-esteem is on a task that does not give them time to reflect on it), and the difference between both (high explicit with low implicit self-esteem or low explicit with high implicit self-esteem). Our results show that youth with autism spectrum disorder report lower self-esteem than youth without autism spectrum disorder when they have reflected on it (explicit self-esteem). And parents of children with autism spectrum disorder report that their children have even lower self-esteem. Implicit self-esteem was the same for youth with and without autism spectrum disorder. Furthermore, we found that within youth with autism spectrum disorder, there was a negative relationship between explicit self-esteem and depressive symptoms, and between implicit self-esteem and externalizing behavior. Taken together, youth with autism spectrum disorder are at risk for developing low self-esteem and when they do they have a higher risk of developing co-occurring problems. Therefore we stress that it is important to measure and improve the self-esteem of youth with autism spectrum disorder, so they develop less co-occurring problems and have a higher quality of life.

## Introduction

Autism spectrum disorders (ASDs) are neurodevelopmental conditions encompassing difficulties with social relationships and communication, often accompanied with repetitive behavior and unusually narrow interests (American Psychiatric Association (APA), 2013). In addition to these symptoms, youth with ASD may notice differences between themselves and typically developing (TD) peers on several other domains. For example, they might have co-occurring language difficulties that cause trouble expressing themselves in language or understanding the language of others, some children show discrepancies among various intellectual abilities, which may impact their academic achievement or have motor difficulties (APA, 2013; Estes et al., 2011).

Children evaluate their achievements and their personality traits by comparing themselves to others and using feedback from others (Mann et al., 2004). In early childhood, feedback is mainly given by parents, but as children grow older, teachers and peers get a more prominent role in children’s lives and their feedback becomes more important for self-evaluation (Harter, 2012). These opinions of, comparisons with, and feedback of others can impact their self-esteem (Harter, 2012; Mann et al., 2004). Given the variety of domains in which youth with ASD might experience they are different compared to TD peers, and the possibility that they get confronted with these differences or are explicitly compared to TD peers, it might be harder for them to maintain a positive self-esteem (Mann et al., 2004).

Self-esteem is defined as the overall evaluation of one’s worth or value as a person (Bailey, 2003; Harter, 2012). Negative or low self-esteem is a non-specific risk factor for mental health and has been associated with a wide range of internalizing and externalizing psychopathology (Cole et al., 2001; Mann et al., 2004; Sowislo & Orth, 2013; Van Tuijl et al., 2014). Estimations show that 70% of children with ASD have at least one co-occurring disorder, whereas 41% have two or more, of which the most prevalent are anxiety disorders, major depressive disorder, oppositional defiant disorder, attention deficit hyperactivity disorder (ADHD), and obsessive–compulsive disorder (Leyfer et al., 2006; Mattila et al., 2010; Simonoff et al., 2008). Moreover, the presence of these co-occurring disorders indicates significantly lower levels of functioning in children with ASD (Leyfer et al., 2006; Mattila et al., 2010). Following the vulnerability hypothesis of self-esteem, low self-esteem might be predictive of the development of co-occurring disorders in youth with ASD (Van Tuijl et al., 2014). Importantly, it has been shown that adults with ASD indicate lower self-esteem than TD adults (Cooper et al., 2017), making it especially pertinent to investigate self-esteem in youth with ASD: if self-esteem in ASD youth is indeed already low, this could be an important aim for treatment to reduce co-occurring psychopathology, enhance functioning, and possibly prevent self-esteem related problems later in life. Therefore, the two aims of this study are to thoroughly assess self-esteem in youth with ASD and to explore the association with their comorbid symptoms.

When measuring self-esteem, self-report questionnaires can be used, which rely on explicit reflection on one’s own self-worth, also called “explicit self-esteem.” Explicit self-esteem is constructed by deliberate, rational, and evaluative cognitive processes and needs motivation, time, and cognitive resources (Strack & Deutsch, 2004). Reported explicit self-esteem might therefore be influenced by, for example, social desirability, attempts to inflate one’s self-evaluation, or problems with self-reflection (Buhrmester et al., 2011). In individuals with ASD, deficits in social cognitive processing have been reported, in particular, with respect to coding of self-referential information (Lombardo et al., 2010). To date, one study investigated explicit self-esteem in youth with ASD, indicating lower explicit self-esteem in ASD compared to TD youth (McCauley et al., 2019). This is in line with studies focused on explicit measures of self-competence, a concept that correlates highly with self-esteem, in which youth with ASD rated themselves lower on social and athletic competence compared to their TD peers, but not lower on their self-worth (Bauminger et al., 2004; Vickerstaff et al., 2007; Williamson et al., 2008). Furthermore, some individuals with ASD are described to show relatively high levels of grandiosity (Abell & Hare, 2005), showing an overestimation of their competences or self-enhancement of their personality traits (Furlano et al., 2015; Lerner et al., 2012; Locke & Mitchell, 2016). All in all, studies on explicit self-esteem and self-competence in youth with ASD show mixed results, which could be a consequence of difficulties in reporting on their self-esteem. For this reason, in this study, we measured self-reported explicit self-esteem as well as an estimation of youth’s self-esteem by their parent.

Dual-process models propose that apart from the explicit, deliberate cognitive process of evaluating the self, there is also an impulsive or more “automatic” process (Strack & Deutsch, 2004), referred to as “implicit self-esteem.” Because implicit self-esteem is an impulsive, automatic process, it has also been defined as “a global self-evaluation that people are unable or unwilling to report.” Even though explicit and implicit self-esteem are believed to influence each other, they show little correlation (Bosson et al., 2000) and so are seen as two different constructs of self-esteem. Therefore, in this study, both explicit and implicit self-esteem were studied.

When cognitive resources are restrained, there are time-constraints, or purposeful reflection is not possible, this “implicit self-esteem” is shaped through automatic processing of affective experiences (Greenwald & Banaji, 1995; Koole & Pelham, 2003; Van Tuijl et al., 2014). However, affective experiences have been shown to be qualitatively different in children with ASD compared to TD children (Hobson, 1986). In addition, youth with ASD may have less affective experiences due to difficulties in forming and maintaining close, affective friendships (Fuentes et al., 2012; Petrina et al., 2014). Therefore, although to date no studies have been conducted on implicit self-esteem of youth with ASD, we expected lower implicit self-esteem in youth with ASD than in TD youth.

A second objective in this study was to investigate the association of self-esteem with co-occurring symptoms in youth with ASD. A previous study on self-esteem in ASD showed that explicit self-esteem of youth with ASD was strongly related to symptoms of depression (McCauley et al., 2019). To date, relationships between explicit self-esteem and co-occurring externalizing symptoms and correlations between implicit self-esteem and co-occurring psychopathology have not been studied in youth with ASD. Previous studies in TD youth show that measures of implicit self-esteem often do not relate to internalizing psychopathology (Bos et al., 2010; Creemers et al., 2012; Van Tuijl et al., 2014).

Measuring both implicit and explicit self-esteem enables us to additionally look at a possible discrepancy between the two. It has been reasoned that “discrepancies in either direction are maladaptive, because they represent deficient integration of self-representations” and are associated with more negative effects on mental health than congruent low or high implicit and explicit self-esteem (Schröder-Abé et al., 2007). For example, a combination of low implicit and high explicit self-esteem, also called “fragile self-esteem,” has been found in individuals high on narcissism (Zeigler-Hill, 2006). The reverse, a combination of high implicit and low explicit self-esteem, also called “damaged self-esteem,” has been associated with internalizing problems in TD individuals (Creemers et al., 2012; Leeuwis et al., 2015; Schreiber et al., 2012). Therefore, in this study, we will explore the association of self-esteem discrepancies and co-occurring symptoms in youth with ASD.

The first aim of this study was to investigate self-esteem in youth with ASD. We expected that (1a) youth with ASD show lower explicit self-esteem compared to TD youth (McCauley et al., 2019) as reported by themselves and their parents and (1b) youth with ASD show lower implicit self-esteem compared to TD youth (Greenwald & Banaji, 1995; Hobson, 1986). The second aim of this study was to explore the association of self-esteem with co-occurring symptoms in youth with ASD. We expected that (2a) measures of self-esteem (implicit and explicit separately) are negatively associated with measures of internalizing and externalizing co-occurring symptoms and (2b) the discrepancy between explicit and implicit self-esteem is related to co-occurring internalizing and externalizing symptoms. That is, we expect that as the discrepancy leans toward a relatively more damaged self-esteem (lower explicit than implicit self-esteem), youth will experience more co-occurring symptoms (e.g. Creemers et al., 2012).

## Methods

### Participants

Thirty-three individuals with ASD and 29 TD peers participated in this study. Both groups were selected based on age (8–16 years) and intelligence quotient (IQ) (>80, see Table 2). IQ was estimated by two subtests (vocabulary and block design) of the Dutch Wechsler Intelligence Scale for Children (WISC-III-NL, Kort et al., 2005). These two subtests correlate highly with full scale IQ (*M* = 100, *SD* = 15; Sattler, 2001).

#### ASD group

The study was promoted by different outpatient mental health institutes of Lucertis GGZ and by the Dutch Association of Autism (NVA/Balans). All individuals that applied for the study had a prior clinical Diagnostic and Statistical Manual of Mental Disorders (4th ed., text rev.) (*DSM*-IV-TR) (Asperger, Classic Autism, or pervasive developmental disorder not otherwise specified (PDD-NOS); APA, 2000) or *DSM*-5 diagnosis of ASD (APA, 2013), determined by an independent child psychiatrist or certified psychologist. Diagnosis was confirmed using the social responsiveness scale (SRS; Constantino & Gruber, 2005; Roeyers et al., 2011). In this study, only youth with a *t*-score of 65 or higher on the SRS were included: five children did not reach this criterion and were excluded. As will be described in more detail below, based on performance on the implicit association test (IAT), three more participants were excluded from further analyses. This resulted in an ASD group of *n* = 25 for analyses.

Within the ASD group, 16 children used medication, of which nine used methylphenidate, one used dexamphetamine, one used aripiprazole, and four used a combination of methylphenidate and aripiprazole. Children who used medication were asked to take medication at least 1 h before assessment to make sure the influence of co-occurring problems (e.g. attention problems) on task performance was minimalized.

#### TD group

TD children were recruited on community schools that are part of the ESPRIT College in Amsterdam. Participants in the TD group did not have any clinical diagnoses and none of the participants had an SRS score above 65 (Constantino & Gruber, 2005; Roeyers et al., 2011). However, as will be described in more detail below, based on IAT performance, five more participants were excluded from further analyses, resulting in a TD group of *n* = 24 for analyses. None of the participants in the TD group used medication.

### Instruments

#### Explicit self-esteem

To measure youth’s explicit self-esteem, we used three Dutch versions of the Rosenberg self-esteem scale (RSES): for children (RSES-C; 8–11 years), adolescents (RSES-A; 12–18 years), and parents (RSES-P). Each version consisted of 10 items, and participants could respond on a scale of 1 (completely not true) to 4 (completely true). RSES scores ranged from 10 to 40, with higher scores indicating a more positive self-esteem. All three versions of this questionnaire consisted of the same items as the original, but the wording slightly differed to match the child’s age. For example, the sentence “I am able to do things as well as most other *people*” was adapted for children into “I am able to do things as well as most other *children*,” for adolescents into “I am able to do things as well as most other *youth*,” and for parents this sentence was preceded by the phrase: “*My child thinks that he/she is* able to do things as well as most other children.” Reported convergent validity (Cohen’s *d* = 0.6–0.8) and internal consistency (Cronbach’s α = 0.91) of the RSES are high (Sinclair et al., 2010). In the current sample, self-reported RSES ratings showed an internal consistency of Cronbach’s α = 0.80 in the TD group and Cronbach’s α = 0.90 in the ASD group. Parent-reported RSES internal consistency was Cronbach’s α = 0.92 in the TD group and Cronbach’s α = 0.93 in the ASD group.

#### Implicit self-esteem

To measure implicit self-esteem, we used the IAT (Greenwald & Farnham, 2000). Greenwald et al. (2003) described the IAT as “a measure of strengths of automatic associations.” Compared to other implicit tasks (e.g. evaluative priming task, affective priming task, and name letter task), the IAT showed the highest internal consistency (Cronbach’s α *=* 0.82; Spearman–Brown corrected split-half reliability = 0.75 to 0.83; Bar-Anan & Nosek, 2014; Krause et al., 2011).

The IAT was programmed using INQUISIT Millisecond 3.0 (2012) software. During the task, participants categorized words into two target categories: “I” and “others” and two attribute categories: “positive” and “negative.” The “I” category consisted of seven words (I, me, myself, self, mine, own, my), as did the “others” category (other, he, she, they, his, hers, theirs). The attribute categories consisted of six positive words (smart, fun, beautiful, kind, winner, and important) and six negative words (stupid, annoying, ugly, unkind, loser, and irrelevant). In line with the original IAT, the task consisted of seven blocks (Greenwald & Farnham, 2000), in which the compatible blocks (“I” and “positive” categories on the same side of the screen) and the incompatible blocks (“I” and “negative” categories on the same side of the screen) were first practiced and then assessed (see Table 1; Greenwald et al., 2003). In all blocks, trials were presented in a random order and the inter-trial interval was 250 ms. To prevent order effects, four counterbalanced sequences were divided randomly across participants (for details, see Table 1) (Lane et al., 2007).

**Table 1.** Procedure self-esteem implicit association test (counterbalance order 1).

Block	No. of trials	Function	Categories assigned to left-key response	Categories assigned to right-key response
1	20	Practice	I	Others
2	20	Practice	Positive	Negative
3	20	Practice compatible	I and positive	Others and negative
4	40	Test compatible	I and positive	Others and negative
5	40	Practice	Others	I
6	20	Practice incompatible	Others and positive	I and negative
7	40	Test incompatible	Others and positive	I and negative

*Note*: To prevent effects of order (Greenwald et al., 1998) and handedness (Yu et al., 2016), blocks were randomly counterbalanced over children, resulting in four counterbalancing sequences: (1) blocks 1, 2, 3, and 4 are compatible with I/positive on the left and blocks 5, 6, and 7 are incompatible, (2) blocks 1, 2, 3, and 4 are incompatible with I/negative on the right and blocks 5, 6, and 7 are compatible, (3) blocks 1, 2, 3, and 4 are compatible with I/positive on the right and blocks 5, 6, and 7 are incompatible, (4) blocks 1, 2, 3, and 4 are incompatible with I/negative on the left and blocks 5, 6, and 7 are compatible.

The relevant labels (“I” and “others” for the target categories and “positive” and “negative” for the attribute categories) were shown in the upper left and right corners of the screen for the entire duration of each block. In each trial, one of the words appeared in the center of the screen. Participants had to indicate as quickly and as accurately as possible to what category the words belonged by pressing the keys “I” (right) or “E” (left) on a standard QWERTY keyboard. Both keys were accentuated on the keyboard with an orange tag. Stimuli remained on the screen until a correct response was given. When the response was incorrect, a red cross appeared on the screen and the participant had to respond again until the right answer was given.

##### Scoring and outliers

IAT scores were calculated using the D-measure with built-in error penalty (D_BIEP_). Greenwald et al. (2003) described D_BIEP_ as “the preferred IAT measure when the IAT procedure allows subjects to correct errors and records latency to the occurrence of the eventual correct response.” The D_BIEP_ measure includes performance on practice blocks, as implicit associations are strongest at the start of an IAT (blocks 3 and 6). Within D_BIEP_, latencies are corrected for individual variability (which in youth with ASD is beneficial, given the increased reaction time variability when children have co-occurring ADHD; Karalunas et al., 2014). Negative scores reflect negative associations toward the self related to others, whereas positive scores reflect positive associations toward the self related to others. A zero-score can be interpreted as a comparable attitude toward self and others. Finally, all children with total error percentages above 20%, or with latencies below 400 ms or above 10.000 ms, will be discarded as the number of errors and latency both affect the D_BIEP_ score (Greenwald et al., 2003).

##### Internal consistency

We calculated the split-half reliability of the self-esteem IAT for both the ASD and the TD group. To this end, we listed all trials by the order of appearance, separately for each stimulus type (I, other, positive, and negative), test block, and participant. Odd and even trials were separated, and two separate D_BIEP_ scores were calculated for each participant (De Houwer & De Bruycker, 2007). Correlations between the separately calculated D_BIEP_ scores of all youth with ASD resulted in a split-half reliability of *r* = 0.69. The split-half reliability of the self-esteem IAT in the TD group was *r* = 0.65. A Fisher *r*-to-*z* transformation showed that the split-half reliabilities in the ASD and TD group were not significantly different from one another (z = 0.23, *p* = 0.82).

##### Neutral IAT

Youth with ASD are known for their executive functioning problems, for example, working memory, inhibition, and task-switching (Demetriou et al., 2018), while the IAT is a cognitive task that uses a switching paradigm. Therefore, to test whether youth with ASD are just as capable as TD peers in doing an IAT, they first completed a “neutral” IAT. This neutral IAT is in every way comparable to the self-esteem IAT (same laptop was used, same syntax to analyze the task output, same positive and negative attributes), except for the target categories “I” and “others” that are replaced with neutral categories: “rooms” (living room, bedroom, kitchen, etc.) and “colors” (yellow, orange, purple, etc.). We compared error rates, latency, and D_BIEP_ between ASD and TD groups, to make sure that results on the self-esteem IAT are not caused by cognitive or executive functioning problems in the ASD group.

Results showed that one child with ASD and three TD children had error rates in excess of 20%. After removal of these participants, no children had latencies below 400 ms or above 10.000 ms. One TD child had a high overall mean reaction time (*SD* = 4.5) causing a skewed distribution, so latency data were log transformed before analyses. Independent t-tests showed that error percentage (*p* = 0.41), mean overall latency (*p* = 0.48), and D_BIEP_ scores (*p* = 0.07) were comparable between groups. This shows that youth with ASD are equally capable of doing an IAT task as their TD peers and results on the self-esteem IAT cannot be attributed to cognitive or executive functioning problems in the ASD group. However, the four children (one ASD and three TD) with high error rates on the neutral IAT were discarded from further self-esteem analyses, in case they did not understand the task or did not take it seriously.

#### Clinical presentations

##### Severity of ASD

Severity of ASD was measured using the SRS (Constantino & Gruber, 2005; Roeyers et al., 2011): a parent-reported questionnaire used to screen for ASD. It is a 65-item scale that measures the ability to engage in emotionally appropriate reciprocal social situations in youth aged 4–17 years. It is rated on scale from 0 (“never true”) to 3 (“almost always true”), with higher scores indicating more problem behavior.

##### Internalizing and externalizing symptoms

Internalizing and externalizing symptoms were assessed using the parent-reported child behavior checklist (CBCL; Achenbach & Edelbrock, 1991; Verhulst et al., 1996). The CBCL consists of 112 questions, and results can be subdivided into an internalizing scale and an externalizing scale. Internal consistency ranges from 0.75 to 0.84 (Achenbach et al., 2003).

##### Depression symptoms

Children self-reported on their depression symptoms using the child depression inventory (CDI; Sitarenios & Kovacs, 1999; Timbremont & Braet, 2002). A self-report measure of internalizing problems was added because child and parent-reported externalizing behavior overlap more than child and parent-reported internalizing behavior (Youngstrom et al., 2000). In this study, internal consistency was α = 0.67 in the TD group and α = 0.87 in the ASD group.

### Procedure

After parents applied for the study, they were called to check inclusion criteria. When inclusion criteria were met, assessment took place at the mental health center where they applied or in the child’s home in a quiet room without others and with as little distraction as possible. Parents and children over 12 years signed informed consent before assessment. Instruments were always offered in the same order during assessments: First, parents were asked to fill in questionnaires and separate from the parent the child completed two WISC subtests and questionnaires. Next, the child did the neutral IAT and subsequently completed the self-esteem IAT.

### Statistical analyses

Data of the self-esteem IAT were checked for outliers: four children had error percentages that exceeded 20% and were removed (*n*_TD_ = 2, *n*_ASD_ = 2), resulting in 24 and 25 participants in the TD and ASD groups, respectively. The normality assumption was violated for parent- and child-reported explicit self-esteem, the CDI, and the internalizing scale of the CBCL (i.e. for the total group and the TD group, not for the ASD group only). In addition, the assumption of homogeneity of variance was violated for all measures of internalizing, externalizing, and depression symptoms. Therefore, in the analyses, parametric tests were used when including only implicit self-esteem or only ASD subjects, whereas non-parametric tests were used in all other analyses. Groups were compared on demographic variables and co-occurring symptoms using independent t-tests or Mann–Whitney U-tests; gender was compared using a chi-square test.

To test our first hypothesis that explicit and implicit self-esteem are lower in youth with ASD, we compared these measures between ASD and TD groups, using Mann–Whitney U-tests (explicit self-esteem) and an independent *t*-test (implicit self-esteem). In addition, we tested whether parents and youth in both groups reported differently about youths’ self-esteem. For this purpose, we conducted a 2 (group: TD, ASD) × 2 (informant: parent, child) repeated measures analysis of variance (ANOVA). The ANOVA is a parametric test, while not all assumptions were met. This should be taken into account when interpreting these results.

To test our second hypothesis that negative explicit self-esteem, negative implicit self-esteem, and the discrepancy between explicit self-esteem and implicit self-esteem are associated with co-occurring internalizing and externalizing symptoms in youth with ASD, we conducted three hierarchical regression analyses with explicit and implicit self-esteem (both centered around their means) in step 1 and the interaction between these two measures in step 2. Outcome variables were depression, internalizing, and externalizing symptoms.

## Results

### Sample description

Groups did not differ in age (*p* = 0.24), IQ (*p* = 0.19), and gender (*p* = 0.12, see Table 2). Parents of youth with ASD reported their children higher on autistic traits (*t*(47) = −13.79, *p* < 0.001, *d* = 3.96), internalizing (*U* = 544.50, *p* < 0.001, *d* = 1.95), and externalizing symptoms (*U* = 542.50, *p* < 0.001, *d* = 1.92) compared to parents of TD children. Similarly, youth with ASD reported more depressive symptoms compared to their TD peers (*U* = 504.50, *p* < 0.001 *d* = 1.44).

**Table 2.** Sample description and group comparison.

	TD (*n* = 24)	ASD (*n* = 25)	Group comparison
	*M* (*SD*)	*M* (*SD*)	
Demographics			
Gender *N* male	10 (41.67%)	16 (64.00%)	*p* = 0.12
Age	12.12 (2.30)	12.89 (2.21)	*p* = 0.24
IQ	111.88 (14.96)	106.00 (15.87)	*p* = 0.19
Severity ASD	26.00 (14.46)	96.96 (20.85)	*p* < 0.001
Co-occurring symptoms			
Internalizing symptoms	6.92 (6.43)	22.52 (11.33)	*p* < 0.001
Externalizing symptoms	4.96 (4.16)	16.00 (7.98)	*p* < 0.001
Depression	5.45 (3.39)	13.52 (7.45)	*p* < 0.001
Explicit self-esteem			
Rosenberg child	33.88 (4.64)	28.76 (6.60)	*p* = 0.007
Rosenberg parent	33.88 (6.42)	23.64 (7.30)	*p* < 0.001
Implicit self-esteem			
D_BIEP_	0.33 (0.39)	0.22 (0.43)	*p* = 0.32

TD: typically developing; ASD: autism spectrum disorder; IQ: intelligence quotient; D_BIEP_: D-score with build in error penalty.

### Group comparison of explicit and implicit self-esteem

**Figure 1.**
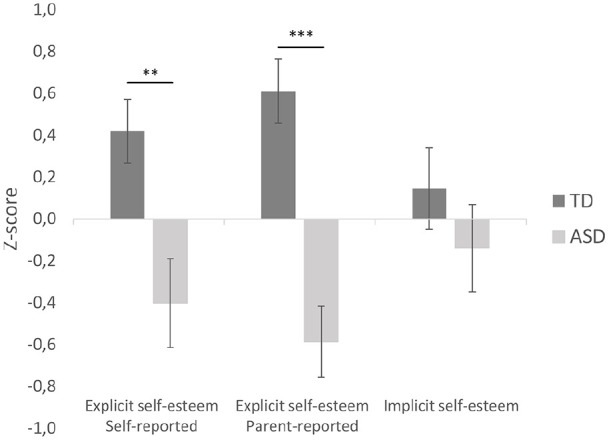
Comparisons of *z*-converted self-esteem measures between youth with ASD and TD (typically developing) peers.

Participants with ASD showed lower explicit self-esteem compared to TD participants according to self-report (*U* = 165.00, *p* *=* 0.007, *d* = 0.84) and parent report (*U* = 96.00, *p* < 0.001, *d* = 1.44; for an overview of self-esteem measures, see Table 2 and Figure 1). In addition, the 2 × 2 repeated measures ANOVA showed a main effect of informant (*F*(47) = 7.63, *p* = 0.008, $\eta_{p}^{2}$ = 0.140), a main effect of group (*F*(47) = 24.40, *p* < 0.001, $\eta_{p}^{2}$ = 0.342), and a group × informant interaction (*F*(47) = 7.63, *p* = 0.008, $\eta_{p}^{2}$ = 0.140). A follow-up Wilcoxon signed ranks test (TD group) and paired *t*-test (ASD group) indicated similar self-esteem ratings indicated by parents (*M* = 28.76, *SD* = 6.60) and youth (*M* = 28.76, *SD* = 6.60) in the TD group (*Z* = −0.366, *p* = 0.714), while youth with ASD reported more positive about their self-esteem (*M* = 28.76, *SD* = 6.60) compared to their parents (*M* = 23.64, *SD* = 1.30) (*t*(24) = 3.63, *p* = 0.001, *d* = −7.33, 95% confidence interval (CI) = [2.21, 8.03]).

Implicit self-esteem did not differ between youth with and without ASD (*t*(47) = 0.100, *p* = 0.32, *d* = 0.28, 95% CI = [−0.19, 0.35]).

### Explicit and implicit self-esteem as predictors of co-occurring symptoms

**Figure 2.**
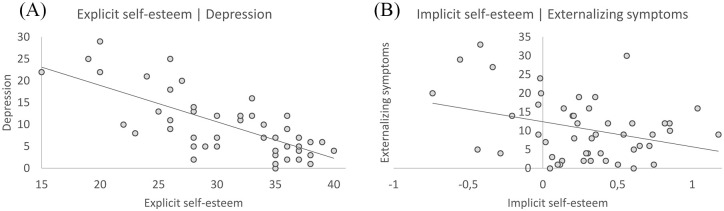
Relationships of self-esteem and co-occurring symptoms in youth with ASD. (A) Negative relationship between explicit self-esteem and reported depression symptoms in youth with ASD. (B) Negative relationship between implicit self-esteem and externalizing symptoms in youth with ASD.

To test whether the explicit self-esteem, implicit self-esteem, and the discrepancy between explicit and implicit self-esteem are related to co-occurring symptoms in youth with ASD, we conducted hierarchical multiple regression analyses on the ASD sample only. Results showed that explicit self-esteem was negatively related to depression symptoms (Table 3a, Figure 2a), whereas implicit self-esteem was negatively related to externalizing symptoms (Table 3c, Figure 2b). A recalculated CDI score (excluding 4 items that may overlap with self-esteem reports) correlated negatively with explicit self-esteem (*r* = −.672, *p* < .001). In the hierarchical regression with this adjusted measure of the CDI as dependent variable, explicit self-esteem remained a significant predictor (*β* = −.820, *t*(21) = −5.125, *p* < .001). The interaction between explicit and implicit self-esteem was not related to co-occurring depression, internalizing, or externalizing symptoms.

**Table 3.** Hierarchical regression analyses: Associations of implicit and explicit self-esteem and the interaction between both with (a) depression, (b) internalizing symptoms, and (c) externalizing symptoms (*N* = 25).

	Δ*R*²	*B*	*SE*	*β*	*p*
**(a) Depression**					
Step 1	0.556				
Explicit self-esteem		−0.894	0.171	−0.793	<0.001*
Implicit self-esteem		3.971	2.646	0.227	0.148
Step 2	0.053				
Explicit self-esteem		−0.967	0.169	−0.857	<0.001*
Implicit self-esteem		2.319	2.721	0.133	0.404
Interaction explicit × implicit		−0.484	0.286	−0.266	0.105
**(b) Internalizing symptoms**					
Step 1	0.040				
Explicit self-esteem		−0.323	0.382	−0.188	0.406
Implicit self-esteem		−0.711	5.919	−0.027	0.905
Step 2	0.004				
Explicit self-esteem		−0.352	0.403	−0.205	0.393
Implicit self-esteem		−1.363	6.478	−0.051	0.835
Interaction explicit × implicit		−0.191	0.681	−0.069	0.782
**(c) Externalizing symptoms**					
Step 1	0.362				
Explicit self-esteem		0.318	0.219	0.263	0.161
Implicit self-esteem		−11.981	3.399	−0.639	0.002*
Step 2	0.003				
Explicit self-esteem		0.301	0.231	0.249	0.208
Implicit self-esteem		−12.388	3.718	−0.661	0.003*
Interaction explicit × implicit		−0.119	0.391	−0.061	0.763

* Bold values signify that analyses give value 0.000. *SE*: standard error.

Depression: step 1—*p*_change_ < 0.001 and step 2—*p*_change_ = 0.105; internalizing symptoms: step 1—*p*_change_ = 0.641 and step 2 *p*_change_ = 0.782; and externalizing symptoms: step 1—*p*_change_ = 0.007 and step 2—*p*_change_ = 0.763.

## Discussion

This study was the first to investigate both explicit and implicit self-esteem in youth with ASD and to additionally test for possible associations of self-esteem with co-occurring (internalizing and externalizing) symptoms in youth with ASD.

### Explicit and implicit self-esteem

Our results showed lower explicit self-esteem in youth with ASD compared to TD youth according to self- and parent report. This is in line with previous studies showing lower self-esteem (McCauley et al., 2019) and lower social and athletic self-competence (Bauminger et al., 2004; McCauley et al., 2019; Vickerstaff et al., 2007; Williamson et al., 2008) in youth with ASD compared to TD youth. Remarkably, parents of youth with ASD were more negative than their children about their child’s self-esteem, whereas in the TD group, parents’ ratings of their child’s self-esteem were similar to children’s ratings. There are some possible explanations for this finding. First, youth with ASD might overestimate their explicit self-esteem, due to deficiencies in processing of self-referenced information (Lombardo et al., 2010). A second explanation could be that the parent reports within the ASD group are overly negative, for example, because parent reports might be based on excessive worrying about their child (Lee et al., 2008). Taken together, since both measures of explicit self-esteem are lower in the ASD compared to the TD group, results indicate that parents and children with ASD agree that the child has low explicit self-esteem, but this is more pronounced in parent ratings within the ASD group.

In contrast to explicit self-esteem, we observed no group differences in implicit self-esteem. This conflicts with our hypothesis that implicit self-esteem would be lower in youth with ASD. The self-esteem IAT was considered reliable in the ASD sample as performance on a “neutral” IAT was similar for ASD and TD youth (i.e. indicating no constraints due to, e.g. task-switching difficulties), and the self-esteem IAT showed similar split-half reliabilities as reported in previous studies (Bar-Anan & Nosek, 2014; Krause et al., 2011). Hence, this result indicates that despite youth with ASD may notice differences between themselves and their peers and have quantitatively (Fuentes et al., 2012; Petrina et al., 2014) and qualitatively (Hobson, 1986) less affective experiences, they feel a similar implicit valuation about themselves as their TD peers. A possible explanation for this could be that, while explicit self-esteem is proven to remain relatively stable over time, implicit self-esteem has been shown to be more state-like (Buhrmester et al., 2011). For this reason, future studies may test implicit self-esteem on various occasions and locations, in order to obtain a state-independent measure of implicit self-esteem. Taken together, this study shows that youth with ASD have lower explicit self-esteem, but similar implicit self-esteem as compared to TD youth.

### Relationship of self-esteem with internalizing and externalizing symptoms

The second aim of this study was to test whether self-esteem was related to co-occurring internalizing and externalizing symptoms in youth with ASD. The current study showed that self-reported explicit self-esteem correlated negatively with self-reports of depression, replicating a previous study on self-esteem and depression in youth with ASD (McCauley et al., 2019). Nevertheless, it should be noted that the measured correlation might be stronger than the actual relationship, as having feelings of worthlessness is part of the diagnosis of depression (APA, 2013). There may have been method-variance in both measures such that individuals who have the tendency to report negatively about themselves might similarly do so on both measures.

Interestingly, this study also showed a relationship between implicit self-esteem and externalizing symptoms in youth with ASD. Previous studies showed that implicit self-esteem was not related to internalizing symptoms in TD youth (Bos et al., 2010; Van Tuijl et al., 2014) and our results add to this previous knowledge by revealing that instead, implicit self-esteem could be related to externalizing symptoms in youth with ASD. This result relates to observations in clinical practice, where children with suspected low implicit self-esteem tend to externalize, to overcompensate their low self-esteem (Attwood, 2003). Especially in adolescence, symptoms of externalizing behavior often increase (Bongers et al., 2004; Maggs et al., 1995; Moffitt, 2017), so gaining knowledge about possible underlying mechanisms of this externalizing behavior is important. As this study is the first to reveal this relationship in youth with ASD, it should be carefully interpreted and future studies should aim to replicate this finding and possible processes underlying this relationship such as the involvement of spontaneous or impulsive behavior (Rudolph et al., 2010).

Furthermore, in contrast to our expectation, the discrepancy between explicit and implicit self-esteem was not related to internalizing and externalizing symptoms in ASD (when correcting for main effects of explicit and implicit self-esteem). Even though previous research related a combination of high implicit and low explicit self-esteem (damaged self-esteem) to internalizing problems in TD youth (Creemers et al., 2012; Leeuwis et al., 2015; Schreiber et al., 2012), in this study, we do not replicate this association in youth with ASD.

All in all, these results support the vulnerability hypothesis of self-esteem: low self-esteem appears to be predictive of the development of co-occurring disorders in youth with ASD such as depression and externalizing behavior (Van Tuijl et al., 2014).

### Limitations and future directions

This study has thoroughly investigated self-esteem in youth with ASD by including both self- and parent-reported measures of explicit self-esteem, and a measure of implicit self-esteem. Nevertheless, several limitations have to be acknowledged. First, our sample is relatively small and we have done multiple analyses. This means that power may have been too low to reveal group differences and it increases the risk on chance findings. Therefore, replication of our results is important, preferably in larger samples. Second, as our sample had a wide age range and was relatively young (*M*_age_ = 12.9) and self-esteem generally decreases from childhood into adolescence (Harter, 2012), this may have confounded our results. Future studies are advised to use a sample with a more specific age range to investigate self-esteem in a given life-phase or to test development of self-esteem between childhood and adolescence in a larger sample. Third, to date, the self-esteem IAT has not been validated in children and adolescents and it is unclear whether implicit self-esteem is already “stable” in this age category given the fluctuating explicit self-esteem (Van Tuijl et al., 2014). Finally, in this study, participants always first completed the explicit self-esteem questionnaires before conducting the implicit association task. Previous studies have shown that this can lead to order effects such that correlations between implicit and explicit self-esteem measures may be higher than when the implicit self-esteem task was performed first (Bosson et al., 2000). Although the order effect in the current sample may be reduced as a result of our participants completing a neutral IAT task in between the two measures of self-esteem, it is important to note that the explicit self-esteem ratings may have influenced the implicit self-esteem measure. The question is, therefore, whether having only one assessment of implicit self-esteem, offered directly after the explicit measure gives the best estimate of how these children perceive themselves in daily life. In future studies it is therefore recommended to measure implicit self-esteem more than once and preferably in a common environment (e.g. at school or at home) and independent of an explicit measure of self-esteem, to be sure of a stable estimate.

### Clinical implications

The results of this study have several clinical implications. First, youth with ASD are at risk for developing low self-esteem. When examining self-esteem in youth with ASD, both the perspectives of the child and the parent seem important: self-reported self-esteem seems like a better predictor of co-occurring problems than parent-reported self-esteem, but youth with ASD may underreport problems as compared to their parents. Therefore, we suggest that the use of multiple informants is valuable. Second, this study showed that low self-esteem is associated with co-occurring depressive symptoms and externalizing behavior in youth with ASD. As these symptoms or even disorders often coincide with ASD (Simonoff et al., 2008), future studies might investigate whether improving self-esteem would be beneficial to reduce co-occurring symptoms. Previous studies in adults with eating disorders, depressive disorder, or schizophrenia showed that cognitive behavioral therapy aiming at improvement of self-esteem, not only improved self-esteem but also improved depressive symptoms (Korrelboom et al., 2009, 2011, 2012; Van Der Gaag et al., 2012). Therefore, it seems important to explore treatment possibilities to improve self-esteem in youth with ASD as well. Even though replication of these results is necessary, this study shows the importance to focus more on self-esteem in assessment and treatment of youth with ASD.
